# Extracting the mean size across the visual field in patients with mild, chronic unilateral neglect

**DOI:** 10.3389/fnhum.2012.00267

**Published:** 2012-10-05

**Authors:** Allison Yamanashi Leib, Ayelet N. Landau, Yihwa Baek, Sang C. Chong, Lynn Robertson

**Affiliations:** ^1^Robertson Cognitive Neuropsychology Lab, Veterans AdministrationMartinez, CA, USA; ^2^Department of Psychology, University of California BerkeleyBerkeley, CA, USA; ^3^Ernst Strüngmann Institute (ESI) in Cooperation with the Max Planck Society, Pascal Fries LaboratoryFrankfurt, Germany; ^4^Vision, Cognition, and Consciousness Lab, Department of Psychology and Graduate Program in Cognitive Science, Yonsei UniversitySeoul, South Korea

**Keywords:** statistical summary, unilateral neglect, attention, visual perception

## Abstract

Previous studies suggest that normal vision pools information from groups of objects in a display to extract statistical summaries (e.g., mean size). Here we explored whether patients with mild, chronic left neglect were able to extract statistical summaries on the right and left sides of space in a typical manner. We tested four patients using a visual search task and varied the mean size of a group of circles within the display. On each trial, a single circle first appeared in the center of the screen (the target). This circle varied in size from trial to trial. Then a multi-item display appeared with circles of various sizes grouped together either on the left or right side of the display. The instructions were to search the circles and determine whether the target was present or not. The circles were always accompanied by a group of task-irrelevant triangles that appeared on the opposite side of the display. On half the trials, the mean size of the circles was the size of the target. On the other half the mean size was different from the target. The patients were not told that this was the case, and no explicit report of the statistics was required. The results showed that when the targets were absent patients produced more false alarms to the mean than non-mean size when the circles were on the left (neglected) side of the display. This finding demonstrates that statistical information was implicitly extracted from the left group of circles. However, summary statistics on the right side were not limited to the circles. Rather it appears that participants pooled the distractors with the target circles, yielding a skewed statistical summary on the right side. These findings are discussed as they relate to statistical summary processing, visual search and segregation of right and left items in patients with mild, chronic unilateral neglect.

## Introduction

When we walk down a crowded street we encounter a scene rich with information. Typically, we form the impression that we have a full representation of our surroundings. However, due to limitations of the visual system, it is unlikely that we formulate a detailed representation of every object in the scene. Instead, we achieve an overall interpretation of the scene. One way that we formulate this “gist” is via statistical summary (see review, Alvarez, 2011). Within almost every visual scene, there are numerous redundancies, and we can gain a quick average summary of similar features in the environment by calculating statistical summaries. Statistical summary of similar sets of objects has been demonstrated in several areas of visual perception. For instance, Ariely (2001) and Chong and Treisman (2003) reported that subjects can judge the average size of circles in a visual display as well as the average size of items grouped together on the right or left side of a display. Similarly, Parkes et al. (2001) reports that subjects can determine the average orientation of items in the visual field. Others have shown that subjects can accurately judge the mean direction of motion (Williams and Sekuler, 1984) and speed (Watamaniuk and Duchon, 1992). More recent work has shown that statistical summary can occur over time as well (Haberman et al., 2009; Albrecht and Scholl, 2010). Statistical summary is used in countless ways and normally serves individuals well. We employ it not only to summarize characteristics of simplistic objects (i.e., geometric shapes), but also to obtain the average walking direction or higher-order face characteristics of a crowd (Haberman and Whitney, 2007; Sweeny et al., 2011; Yamanashi Leib et al., 2012).

In addition, summary statistics are dependent on accurate grouping of items within the visual field. For instance, if a sweet shop captures a person's attention while walking down a crowded street, statistical summary processes may extract the mean color and shape from the storefront display (brown and square). This information could help lead him/her to the conclusion that the store is selling chocolate as opposed to jelly beans. Simultaneously, the visual system may extract summary statistics from objects outside the focus of attention (i.e., the adjacent clothing store). Imagine if the shape and color of distractors (clothing) were averaged with the shape/color of the target (candy). The resulting summary statistics would be distorted. Fortunately, typical perceivers can successfully extract the mean from different groups of objects presented simultaneously. For instance, Chong and Treisman report that subjects can create separate ensemble statistics for groups of differently colored circles and/or circles that are clustered in different spatial locations (Chong and Treisman, 2003, 2005).

Statistical summary mechanisms benefit visual perception in typical populations—but could also potentially be advantageous for patients with attentional deficits. In explicit experimental tasks, unilateral neglect patients are impaired in attentional search on one side of space (Eglin et al., 1989; Behrmann et al., 1997; Esterman et al., 2000; Laeng et al., 2002; Pavlovskaya et al., 2002). Within daily life, these attentional impairments become evident as neglect patients may neglect to eat food from one side of the plate, forget to dress one side of their body, or fail to draw one side of an object (Husain and Rorden, 2003). Although attentional search is degraded, other perceptual mechanisms remain intact. For instance, organizational processes such as grouping or completion (Brooks et al., 2005) across the right and left sides of a display are relatively unimpaired. Additionally, many priming tasks indicate that neglect patients can be implicitly cued by stimuli presented on the left (neglected) side of space (Marshall and Halligan, 1988; McGlinchey-Berroth et al., 1993). We are interested in exploring whether statistical summary, a process that can occur implicitly (Ariely, 2001; Haberman and Whitney, 2007; De Fockert and Wolfenstein, 2009) is similarly spared in neglect patients. If statistical summary mechanisms are spared, this may allow patients to gain an implicit, unitized percept of their surroundings, despite the fact that attentional search mechanisms are degraded. Statistical summary confers multiple benefits to visual perception including: increased precision, information compression, and rapid updating of working memory (Alvarez, 2011; Brady et al., 2005). Such benefits could be especially useful to unilateral neglect patients, who receive limited benefits from explicit attentional search.

To explore this question, we designed an experiment that investigated whether patients with very mild chronic signs of neglect and psychophysical evidence of continued left sided attentional deficits extract statistical summary under implicit conditions. In brief, we presented a target circle centrally, then asked patients search for the target circle size within a multi-circle display with distractors. Importantly, on half the trials, the mean size of the circles was the size of the target. On the other half the mean size was different from the target. We predicted that if patients extract summary statistics, they will form a clear mental representation of the mean circle size within the search display. This mental representation should trigger patients to falsely report that the mean target size is present in the search display—even when it is absent. Thus, there will be an increase in false alarms when the searched target is the mean size of the display. This method of implicit mean detection has been successfully used in numerous statistical summary experiments (Ariely, 2001; Treisman, 2006; Haberman and Whitney, 2007; De Fockert and Wolfenstein, 2009). We adopted the task reported in Treisman, 2006 to accommodate unilateral neglect patients. Specifically, the multi-circle display was shown either on the left or the right side of the screen. It was always accompanied by a group of distractor triangles on the opposite side of the display. In this way, we could examine whether patients could reject these triangles in responding to the circular array. If they could, false alarms on the right and left should show the same pattern of results (i.e, more FAs to the mean than non-mean). However, if the distractors on the left were pooled with the circular array on the right, then the mean would be distorted and a different pattern of results could occur.

## Materials and methods

### Participants

We tested four patients with chronic unilateral left neglect (three males and one female). Three were mildly impaired on only the line bisection and cancellation task from the standard SCAN test for spatial neglect (McGlinchey-Berroth et al., 1996).

#### Line bisection task

The line bisection task involves showing the patient a horizontal line (centrally presented) on a piece of paper. Patients are asked to write a mark in the exact center of the line. Deviation from the right of center may indicate left neglect. Deviation is measured in centimeters.

#### Cancellation tasks

In the cancellation tasks, patients are presented with 16 letters, symbols, or lines scattered across a piece of paper. Symbols and letters are presented with distractors; lines are not presented with distractors. The patient is asked to cross out a target letter, symbol, or line. The patient is given unlimited time to complete the task and verbally indicates completion. Unilateral neglect patients often fail to cross out items on one side of the page because of impaired attention. The total number of missed items is summed, and items missed on both sides are excluded from the calculation. Our patients completed six cancellation tasks in total, each with 16 targets. One patient did not complete the SCAN and was referred to us by a rehabilitation specialist who noted neglect of left sided information. Diagnosis of unilateral neglect was made by an optometrist and confirmed by the psychophysical conjunction task that we administered prior to testing (reported in Table 2). Some patients took the SCAN during the acute stages of neglect (with scores ranging from 2–9 items missed in the cancellation task and deviations of 0.95–11.35 cm from center in the line bisection task) Table 1 reports age, years post onset, and the scores from our patients most recent SCAN tasks. In order to investigate the role of attention in statistical summary processes we included patients that exhibited attentional biases in one hemifield.

**Table 1:** **Patient age and SCAN scores are presented below**.

**SCAN scores**				
**Participant**	**Age**	**Onset prior to testing**	**Cancellation (No. of missed/16)**	**Line bisection (cm to right)**
UNP1	50	5 years	3	1.40
UNP2	68	3 years	1	1.85
UNP3	50	1 year	^*^	^*^
UNP4	77	1 year	2	3.20

* This participant did not take the SCAN.

At the same time, including patients with a moderate unilateral bias allowed the assessment and comparison of performance on both visual fields.

**Figure 1:**
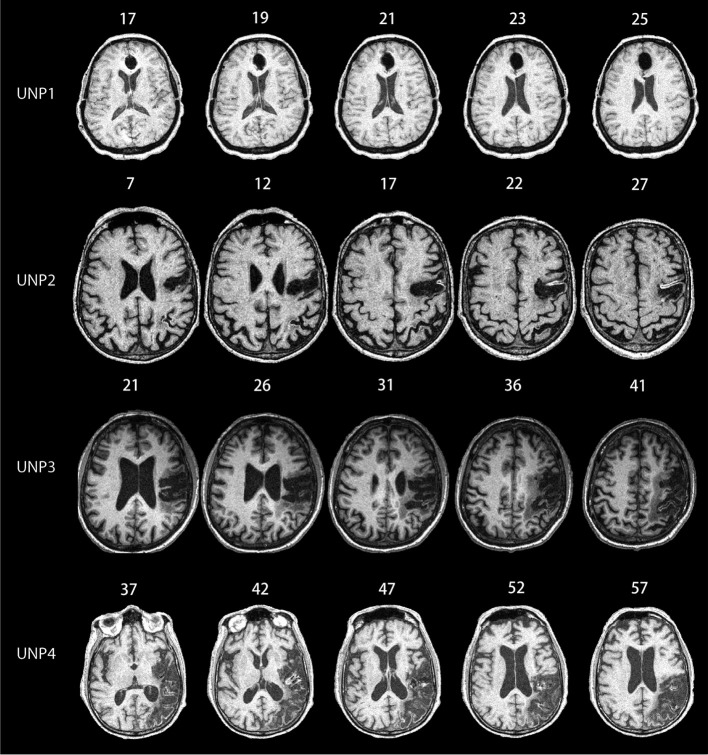
**MRI scans for all of the unilateral neglect patients (UNPs) included in the study.** Numbers listed above each picture depict the MNI coordinates. UNP1 has lesions in the anterior cingulate, orbitofrontal, and thalamic regions. UNP2 has lesions in the frontal, parietal, and occipital regions. UNP3 has lesions in the posterior frontal and anterior parietal regions. UNP4 has lesions in the parietal regions and temporal regions, extending into the temporal-occipital junctions. We include a figure depicting slice regions for each participant in the Appendix, Figure A1.

**Figure A1:**
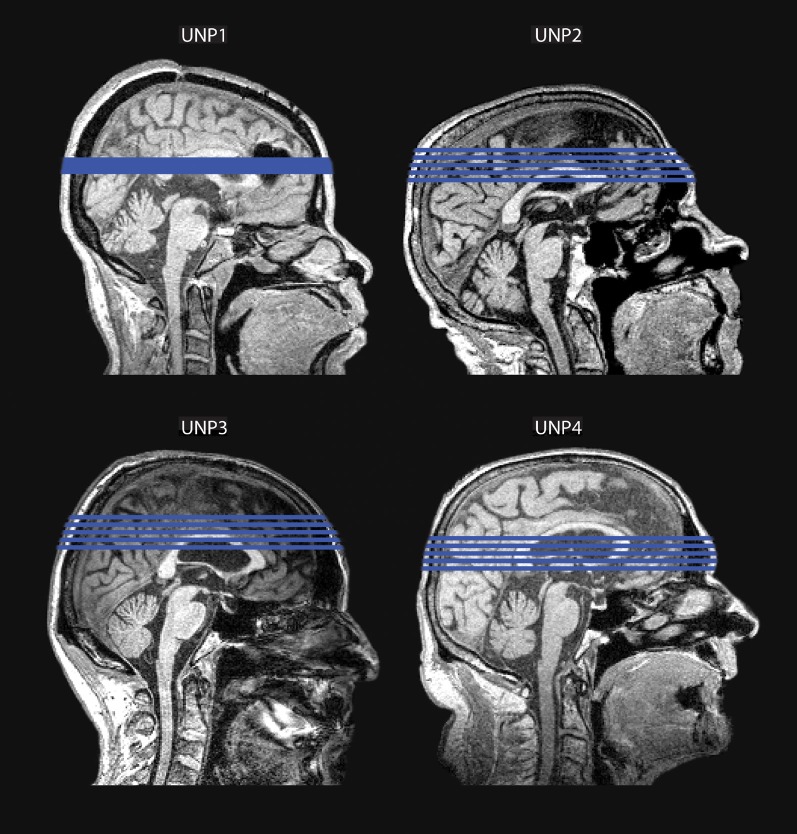
**This figure shows the slice regions (in blue) for all four unilateral neglect patients**.

Previous studies have shown that patients who have minimal signs of neglect on paper and pencil tests, or have never been diagnosed with neglect, will still show significant signs of lateralized impairment during attentionally demanding computerized tests (e.g., List et al., 2008; Bonato et al., 2010, 2012). Thus, the main screening measure was based on a psychophysical task developed to test for chronic signs of neglect [described below (List et al., 2008)]. Note that all four patients were at least 1 year post at the time of testing. Radiological images of lesions for each patient are shown in Figure 1. (Slice regions are provided in Figure A1). Patient 1 had surgery for an aneurysm in the anterior communicating artery resulting in a relatively small anterior cingulated lesion. Vasospasm resulted in significant right orbitofrontal damage and smaller lacunars in the right lateral thalamus and deep inferior basal nucleus of Minert. The remaining patients all had infarct to the territory of the right middle cerebral artery. Patient 2′s lesion included frontal, parietal and occipital regions. Patient 3′s lesion included posterior frontal and anterior parietal regions. Patient 4′s lesions included parietal and temporal regions, extending into the temporal occipital junction. All patients were diagnosed with acute unilateral neglect shortly after being hospitalized. All patients gave informed consents approved by the Internal Review Board at the VA, Northern California Health Care System.

### Psychophysical testing for chronic neglect

Each patient first completed a computerized conjunction search task designed to measure attentional search times (see Treisman and Gelade, 1980). This test was altered to detect symptoms of chronic attentional deficits in neglect using a psychophysical staircase procedure (List et al., 2008). In this task, patients view a screen of colored geometric shapes, and are asked to verbally respond whether there is a red square (target) among distractors. The distractors include a combination of blue squares, red circles, and red/blue triangles. The target is randomly presented on either the left or right side of the display, and exposure times for each trial are adjusted according to participants' performance. In this adaptive staircase procedure, the display is initially presented for 2000 ms. Exposure time decreases when patients correctly identify the target and increases when patients incorrectly identify the target. The staircase is thresholded to produce 75% correct performance both on the left and right sides of the display. Consistent with chronic neglect measures (List et al., 2008), our patients required significantly longer viewing durations when the target was displayed on the left (mean = 826 ms) compared to the right (mean = 483.5 ms) side of the screen, *t*_(3)_ = 4.175, *p* < 0.025. See Table 2 for individual response times in each hemifield.

**Table 2:** **This table shows the exposure duration needed for patients to detect targets displayed the left or the right side of the screen**.

**Threshold display times (ms)**		
**Participant**	**Left target**	**Right target**
UNP1	388	182
UNP2	620	404
UNP3	704	154
UNP4	1592	1194

### Size discrimination task

Having established symptoms of unilateral attentional neglect in each participant, we proceeded to measure their size discrimination ability. This was important, as the task required in the statistical summary experiment is based on size judgments. During this task, the participant was shown a circle for 1000 ms in the middle of the screen followed by a second circle until response. We asked patients to report which of the two circles was bigger (by indicating “first” or “second”). Importantly, the circles sizes were identical to those used in the main experiment (see below). There were 20 trials in total. All patients accurately discriminated circle size with a performance of 90% or above.

### Statistical summary procedure

**Figure 2:**
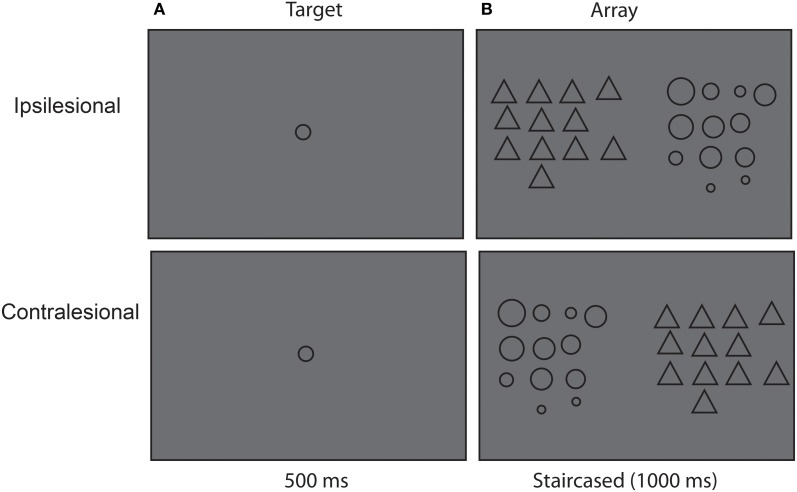
**Experimental procedure. (A)** First patients viewed a target circle and were asked to remember its size. **(B)** Next, patients viewed a display of 12 circles and distractor triangles. The position of the circles and triangles varied between trials, such that sometimes the circle were presented on the right side and the triangles were presented on the left side and vise versa.

Subsequently, each patient participated in the main statistical summary portion of the experiment. Each trial began with fixation (500 ms). Next, we showed the patient a single target circle in the center of the screen for 500 ms that varied in size from trial to trial. This was followed immediately by a search display containing a group of circles on one side and a group of task-irrelevant triangles on the other. The patients were instructed to ignore the triangles and to indicate whether the target size was present or absent in the group of circles. Patients verbalized “yes” for target size present or “no” for target size absent. The examiner keyed in each response on an external keyboard. Importantly, half of the targets matched the mean size of the circles group within the search display, whereas half did not. Figure 2 depicts a schematic example of a target circle (on the left panel in the figure), which was randomly chosen from eight possible sizes with equal probability (diameter 0.98°, 1.12°, 1.26°, 1.29°, 1.40°, 1.47°, 1.66° and 1.84°). Figure 2 also depicts a schematic search display (circles on the right side in the figure) containing 12 circles presented on either the left or right side of the display. Each circle in the display was randomly selected between 0.60° and 2.72° with two constraints. First, their mean size had to match with the pre-defined mean size of the display in the half of trials and they did not match in the other half. Second, neighboring sizes of the target (both mean and non-mean sizes) should have the same distance from the target. This method of choosing sizes was adapted from Treisman (2006) in a study of normal statistical size processing. The triangles were similar in size to the largest circle but their location was jittered between trials. Resolution of the screen was 1024 × 768 with a 60 Hz refresh rate. On half the trials the target size was present, whereas in the remaining half, target size was absent. The exposure of the search display began at 1000 ms, as determined by the size discrimination procedure and was staircased to produce 70% correct performance across all conditions on average. Successful performance on two trials decreased display exposure by 66.67 ms, whereas unsuccessful performance on one trial increased display duration for 66.67 ms. The number of trials varied slightly, as we deleted trials if the patient was inattentive or made eye movements (mean no. of trials = 125, range = 117–128). Thus, on average there were 62 targets present and 62 target absent trials: 31 on the left and 31 on the right. Fifteen of each was the mean and 15 were the non-mean size.

Prior to the experiment, practice trials were given until subjects indicated they were comfortable with the task/instructions (but no less than 10 trials). All practice trials included feedback. Incorrect answers were followed by a brief high-pitched tone, whereas correct answers were followed by an absence of sound. There was no feedback in the experimental trials.

## Results

### Hits rates

**Figure 3:**
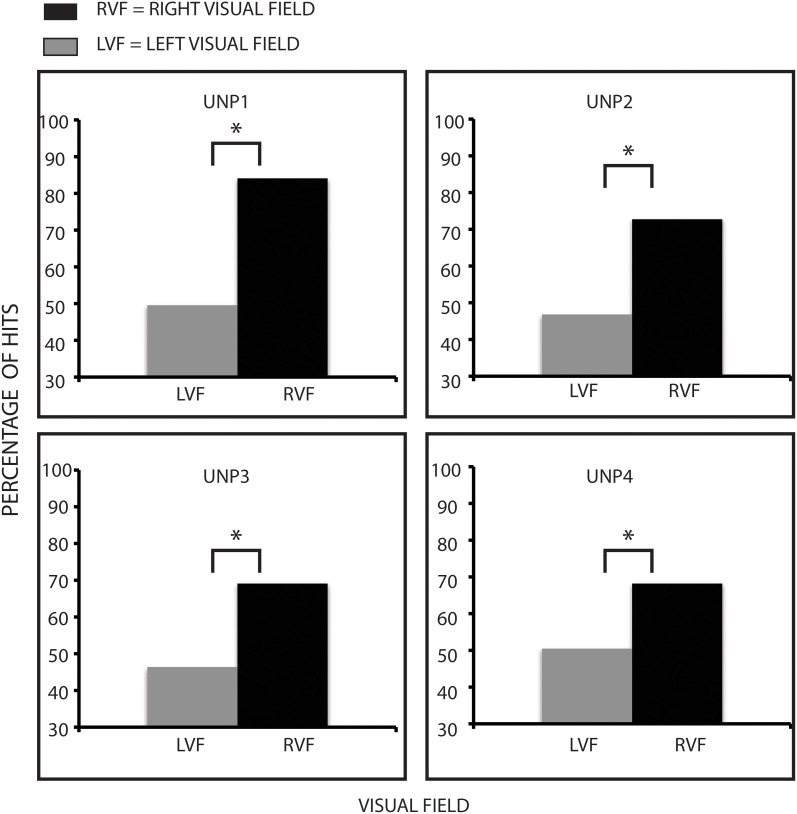
**Hit rate patterns for participants.** Hits rates are lower on the left side compared to the right side, indicating that neglect patients have difficulty performing visual search. ^*^Denotes statistical significance of at least *p* < 0.05.

We calculated the hit rate for mean and non-mean trials for each participant in each hemifield. Hit rate performance was then subjected to a 2 × 2 ANOVA for the group as a whole with the following factors: Hemifield (Right and Left) and Target Statistic (Mean and Non-Mean). There was a significant main effect of Hemifield [*F*_(1, 3)_ = 54.857, *p* = 0.005, η*p*^2^ = 0.948], with patients performing better on the right side compared to the left side. There were no other main effects or interactions that even approached significant levels. We formally assessed whether hemifield differences were significant for each participant by using a bootstrapping technique (200 iterations per participant). Each bootstrapped sample is permuted to simulate variations that may occur over a greater number of trials (see Efron, 1986). We compared the distribution of bootstrapped samples using the Kolmogorov-Smirnov test (Kolmogorov, 1933; Smirnov, 1948). This non-parametric test evaluates whether boot sample mean distributions for two conditions are from the same continuous distribution or whether the samples are from two different continuous distributions. All k statistics show that participants' hit performance was significantly better for detecting targets on the right compared to the left right side (UNP1, *k* = 0.99, *p* < 0.001; UNP2, *k* = 0.88, *p* < 0.001; UNP3, *k* = 0.85, *p* < 0.001; UNP4, *k* = 0.76, *p* < 0.0001). See Figure 3.

### False alarm rates

**Figure 4:**
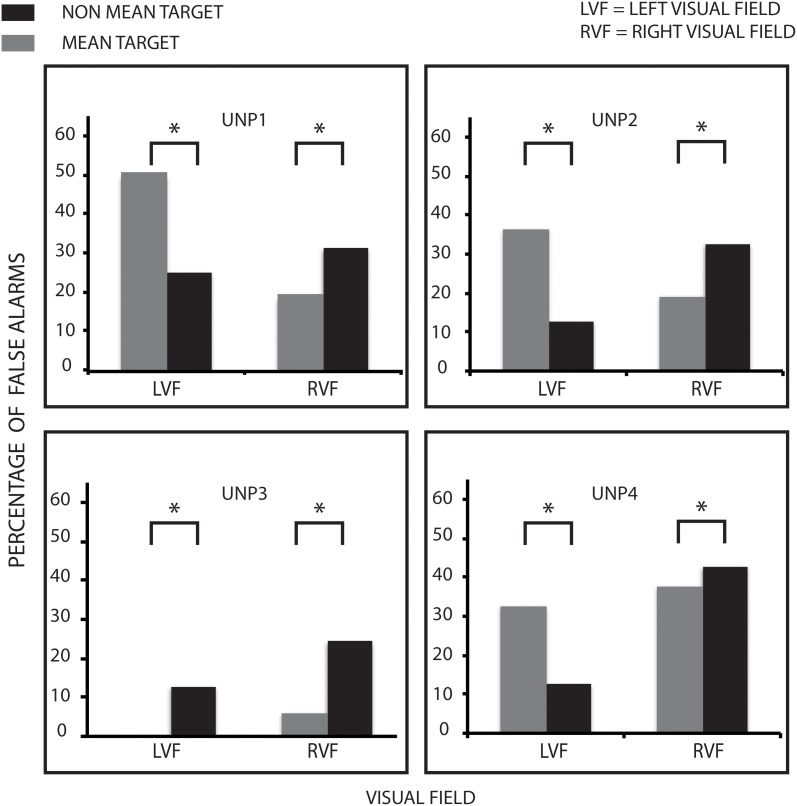
**False alarm rates for participants.** False alarm rates are higher to the mean compared to the non-mean target on the left (neglected) side, mirroring the expected performance for neurologically intact participants. However, false alarm rates on the right (non-neglected) side exhibit the opposite pattern. ^*^Denotes statistical significance of at least *p* < 0.05.

We calculated the false alarm rates for mean and non-mean trials for each participant in each hemifield. The 2 ×2 ANOVA on the group as a whole showed a significant interaction between Hemifield and Target Size, [*F*_(1, 3)_ = 13.252 *p* = 0.036 η*p*^2^ = 0.815] but no significant overall main effects. Motivated by this interaction, we then examined each patient's data with the same bootstrapping method as described above. 3 of the 4 patients showed greater false alarms to the mean compared to the non-mean on the left side with 1 having very few false alarms and reversing (UNP1, *k* = 0.71, *p* < 0.0001; UNP2, *k* = 0.77 *p* < 0.0001; UNP3, *k* = 0.84, *p* < 0.0001; UNP4, *k* = 0.61, *p* < 0.0001). Conversely all four patients showed greater false alarms for the non-mean compared to the mean on the right side (UNP1, *k* = 0.47, *p* < 0.0001; UNP2, *k* = 0.45, *p* < 0.0001; UNP3, *k* = 0.75, *p* < 0.0001; UNP4, *k* = 0.27, *p* < 0.0006). Figure 4 shows the false alarm rate for each patient.

## Discussion

### Visual search

Consistent with the neglect literature and visual search, patients' performance (as measured by hit rates) was worse on the left than the right side. All patients exhibited this pattern with hit rates on the left close to chance levels, consistent with unilateral neglect (see Figure 3). Each patient saw the search display for a different amount of time, as our goal was to keep the accuracy rate across the whole display as close to 70% as possible. We did this using the staircase method to adjust the duration of the search display throughout the experiment. The main question regarding visual search was whether performance differed between the right and left sides, and this was the case. When the left target was present, hit rates on the left hovered around chance performance (i.e., we were able to induce severe left neglect at these short stimulus presentations), while on the right it was well above chance performance. The difference in search between the two sides is consistent with fast and more efficient attentional deployment on the right compared to the left side of the display. This pattern is also consistent with left hemi-extinction, the less dramatic cousin to left unilateral neglect. When items occur on both sides of a display, left items are more likely to be missed. Since every trial had a group of irrelevant triangles opposite the circles, both sides were always filled with stimulation. As intended, these triangles appear to have attracted attention when they were on the right side (Eglin et al., 1989).

### Statistical summary

However, the processes governing extraction of statistical summary appears to differ from those governing individuation of an object in search. Although this distinction has been proposed before in the literature with typical observers (Ariely, 2001; Chong and Treisman, 2003; Haberman and Whitney, 2007), there is no strong evidence for it in neglect patients. The present results demonstrate dissociation between performance measures for visual search and those for statistical summary. These can be seen most robustly in the false alarm rates on the left and right sides. When the target was absent most of the patients were more likely to say it was present when the average of the circle was the mean than when it was the non-mean. The opposite was true when the circles were on the right for all four patients (more false alarms to the non-mean than mean). If we consider the display as a whole, the reasons for this pattern become clearer. Recall that during every trial, distractor triangles must be discounted in order for participants to form a statistical summary, consistent with the target size. If the triangles are not rejected, they will contribute to the estimate of the mean size—and skew the statistical summary. Returning to the scenario presented in the introduction, the neglect patients may be “pooling the clothes in the adjacent store window with the candy.”

### Distractors

This hypothesis is consistent with previous work on unilateral neglect suggesting that information presented on the neglected side is inappropriately filtered. For instance Kim (1997) used a negative priming paradigm in which two letters overlapped. One letter was the target (e.g., the red one), while the other letter was the non-target or distractor. Negative priming is indicated by a slower response to a distractor letter when it appears as a target on a subsequent trial. Results in patients with unilateral neglect demonstrated that negative priming is normal when displays are on the right side, but positive priming appears when displays are on the left side, indicating that the distractor letter was not inhibited. If this is the case, distractors in a statistical summary test may significantly impact extraction of the mean. Specifically, if information on the neglected left side is improperly filtered, the presence of distractors should compromise statistical summary on the right side of the display—and this is what we found.

Figures 3 and 4 together suggest that statistical encoding took place implicitly even though controlled (explicit) attentional search of the left side was reduced. On the right side, the pattern of results did not support statistical processing. Indeed, the pattern of FAs was reversed (more FAs when the target was non-mean). We performed non-parametric tests on the right side to further statistically examine these results. Again, all patients showed a significantly greater effect to the non-mean on the right side. This skewed pattern of FAs indicates that statistical processing in the right hemifield was disrupted. One interpretation of this result is that distractors could be rejected from the summary statistic when presented on the right side but not when presented on the left side.

### Impact of distractors

**Figure 5:**
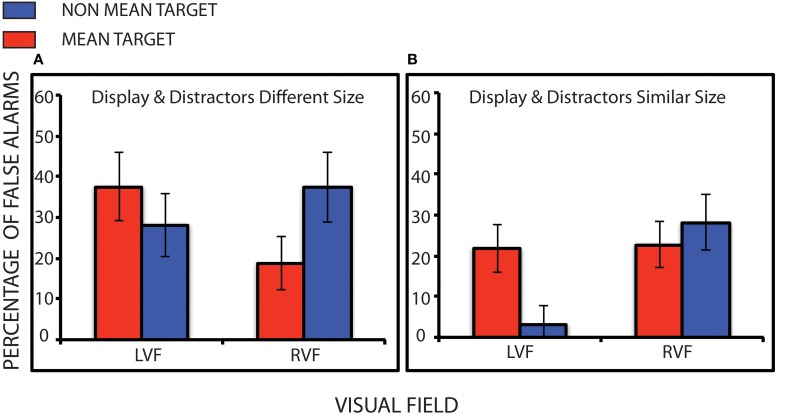
**False alarm rates for patients during trials when distractor and display were very different in size (A) and trials when distractor and display were more similar in size (B).** Error bars represent the standard deviation of the bootstrapped samples. Note: In no case were the distractors and targets the same size. In panel **(A)**, differences between false alarms to the mean and non-mean on the right side are large (and mirror the original findings), suggesting that distractors are influencing statistical coding. Whereas, in panel **(B)**, there is minimal difference between the mean and non-mean targets on the right side, suggesting that distractors are minimally impacting statistical coding.

In order to further explore whether distractors presented on the left side are “encroaching” into the target pool (and thereby resulting in an erroneous statistical summary), we compared how distractor size affected performance when search display circles were smaller or larger. Importantly, distractor triangles are always the same size (the base of the triangle was similar in size to the largest circle diameter). However, circles within the search display varied in size, rendering distractors either more or less close to the circles mean size. This allowed us to observe whether the pattern of false alarms was affected by the distractors. The false alarm pattern presented in Figure 5 provides provisional support that subjects included distractors into their judgment of the mean—when distractors were presented on the left side. Data from all participants are concatenated to increase the number of trials evaluated (as each participant saw only eight trials with small circles and eight trials with large circles). When distractors and display were different in size (Figure 5B), the pattern of false alarms is distorted (more false alarms to the non-mean compared to the mean). However, when distractors and display were similar in size (Figure 5A) the distorted pattern decreased. This is a small but not unexpected difference (given the small difference in mean size), and this would be the expected pattern if the triangles on the left were pooled with the circles displayed on the right.

### Summary

Previous research indicates that unilateral neglect causes widespread disruption in attention, visual working memory, and spatial representation (Husain and Rorden, 2003; Malhotra et al., 2005). These impairments negatively impact daily functioning in everyday life, resulting in problems with navigation, driving, reading, etc. While much is known about the disruptions in controlled attention and visual search, the role of statistical summary has only begun to be tested (Pavlovskaya et al., 2010, 2011). Here we tested patients who had recovered from clinical signs of neglect but continued to show neglect of the left side in a visual search paradigm and when display times were limited. There is some evidence from studies of patients with the neuropsychological diagnosis of Balint's syndrome that statistical summaries may be calculated from unattended information in the visual field (Riddoch and Humphreys, 1987; Demeyere and Humphreys, 2007; Demeyere et al., 2008). However, to our knowledge this is the first study to explore how the extraction of statistical summary may be affected by distractors in bilateral, grouped displays. This is a particularly relevant question because statistical summary in the real-world rarely, if ever, occurs without distractors.

Our results show that patients with chronic neglect (as measured psychophysically) successfully segregated distractors on the right when targets were on the left side. They showed the expected pattern of statistical summary on the left—despite the fact that these patients allocated limited attention to this side (i.e., were at chance explicitly detecting the target). This result supports and expands Pavlovskaya et al.'s findings (2010, 2011), which suggested that neglected items contribute to explicit statistical summary estimates. Moreover, both findings reinforce Alvarez and Oliva's previous work with healthy normal participants, showing that statistical summary occurs even with reduced attention (Alvarez and Oliva, 2009; Joo et al., 2009; Haberman and Whitney, 2011) but extend this work by showing that statistical summary can be successfully performed by patients with unilateral attentional deficits. However, on the right side, patients showed more false alarms to targets that were the mean size compared to targets that were the non-mean size. One interpretation of this result is that neglect patients pooled distractors on the left side with targets on the right side causing the resulting statistical summary to be based on the display as a whole and thus distorted.

### Future directions

These results imply that within real-world settings, neglect patients' ability to statistically summarize different sets of objects may be compromised. Future studies should further investigate whether altering the distractor features reduces the negative impact upon the statistical code in the right hemifield. For instance, our distractors, while different in shape, shared similar outline/filler color with the targets. It is possible that increasing the contrast in target outline/filler compared to distractor outline/filler will reduce pooling of targets and distractors. Further exploration of how distractor/target congruency interacts with statistical summary may yield a greater understanding of the neglect phenomenon and potentially contribute to rehabilitative programs.

Interestingly, the different pattern of performance between hits and false alarms suggests that statistical summary processes are distinct from object individuation. When the target was present, the patients exhibited the expected pattern of performance during visual search (poor performance on the left/better performance on the right). Whereas, when the target was absent, statistical coding dominates. This pattern reinforces previous research showing that object individuation operates independently from statistical summary mechanisms. For instance, neurologically intact participants can perform at chance when asked to individuate objects, yet, are still remarkably accurate in statistically summarizing across objects (Ariely, 2001; Haberman and Whitney, 2007; De Fockert and Wolfenstein, 2009; Haberman et al., 2009; Alvarez, 2011). Additionally Haberman and Whitney (2011), using change blindness paradigms, demonstrate that statistical summary occurs independent of change localization. We reinforce and extend these findings by showing that when object individuation does occur, it is distinct from statistical summary performance.

Our findings also raise interesting questions about how attention influences statistical summaries within normal populations. Chong and Treisman (2005) found that neurologically intact participants can successfully segregate items into different groups to produce separate statistical codes. However, they also found that the individual group averages were nonetheless affected by the overall average of both groups. Further studies should explore how distractors interact with the formulation of the statistical code, and specifically how reduced attention affects the filtering of distractors. Under impoverished attentional conditions (e.g., divided attention, peripheral viewing), can normal perceivers successfully segregate distractors?

## Conclusion

In conclusion, fundamental statistical summary abilities on the left side remained intact in patients who presented with unilateral neglect that had substantially abated but continued to be robust on psychophysical tests. Under conditions that amplify neglect, the patients did summarize the statistics in a display. However, within the real-world, where targets and distractors are equally present within the visual environment, it is important to be able to pool information within different sets. We show for the first time that patients' with left sided attentional deficits, while not interrupting the averaging process *per se*, nonetheless alter summary statistics on the right side. Abnormal statistical coding may substantially affect patient functioning, as statistical summary operates on many levels of visual processing integral to daily life. Research has shown that it contributes to low-level processing (simple shapes), high level processing (faces and other complex stimuli), and visual working memory (Alvarez, 2011). Our work here, with chronic unilateral neglect patients, indicates that distractors encroach into summary coding of target displays, and the statistical summary fails to reflect veridical statistics of the target group. Such distortions may adversely affect the visual analyses of complex scenes in the real-world for such patients.

### Conflict of interest statement

The authors declare that the research was conducted in the absence of any commercial or financial relationships that could be construed as a potential conflict of interest.
